# Evaluating the antagonist effect of naltrexone implant via opioid challenge tests with escalating doses of hydromorphone injection in former heroin dependent patients

**DOI:** 10.3389/fpsyt.2025.1441598

**Published:** 2025-01-30

**Authors:** Wei Qu, Xuyi Wang, Chongyang Dong, Tao Zhang, Shugui Yin, Zhijun Sun, Shiqiang Wang, Anni Guo, Wei Hao

**Affiliations:** ^1^ Research Department, Shenzhen Sciencare Pharmaceutical Co., Ltd., Shenzhen, China; ^2^ Institute of Mental Health of the Second Xiangya Hospital of Central South University, National Medical Center for Mental Disorders, Changsha, China; ^3^ Peking University Clinical Research Institute, Peking University First Hospital, Beijing, China

**Keywords:** naltrexone, opioid blockage, opioid challenge, hydromorphone, long-acting naltrexone implant (NTX-IMP)

## Abstract

Opioid dependence is a serious, life-threatening condition with severe social impacts. Naltrexone (NTX) can weaken the effect of opioids and effectively reduce opioid self-administration, discrimination, and opioid-induced subjective effects, and the oral dosage form has been approved for the treatment of opioid dependence. However, the effectiveness of oral naltrexone as an opioid antagonist has been limited due to poor patient adherence. A long-acting formulation in the form of naltrexone implant (NTX-IMP) with a five-month duration of action may address this issue and improve outcomes. This study (trial registration number: CTR20181954) aimed to evaluate the effect, safety, and pharmacokinetics of NTX-IMP in agonist effects via hydromorphone challenge test, and to determine optimal dosages for future research. Thirty-one former opioid-dependent individuals were randomized to the 0.9g or the 1.5g NTX-IMP group. All subjects exhibited significant antagonistic effects during hydromorphone challenge test. Calculation of slope between VAS score or pupil diameter and hydromorphone dose suggested a stronger antagonistic effect in the 1.5 g group. Pharmacokinetic data suggested that effective plasma naltrexone concentration (≥1ng/ml) was detected from the third day for over 148 days, with higher concentration and longer duration in the 1.5 g group. All subjects tolerated NTX-IMP well. The findings indicate that the NXT-IMP effectively blocks the agonistic effects of hydromorphone in a dose-dependent manner.

## Introduction

Opioid dependence is a serious, life-threatening condition with severe social impacts such as increased mortality, poor social functioning, and crime. Over the past decade, the global incidence of this condition has increased. Despite growing treatment rates, many patients remain untreated (1). Agonist maintenance treatment, using methadone or buprenorphine, provides public health benefits including reducing illicit drug use, lowering HIV rates, and improving patient functioning. However, 122 of 192 UN countries limit or don’t offer this treatment due to a preference for opioid-free methods or concerns about dependence and illegal drug use. Meanwhile, this therapy may not be suitable for certain demographics like young people, new patients, and professionals prohibited from opioid use (2).

Naltrexone is an opioid receptor antagonist that binds to μ opioid receptors 20 times more potent than morphine. It can block or weaken the effect of opioids and effectively reduce opioid self-administration, discrimination, and opioid-induced subjective effects (3–5). Oral dosage forms of naltrexone have been approved by the US Food and Drug Administration (FDA) for the treatment of opioid dependence since 1984 and alcohol dependence since 1994. However, the clinical efficacy of oral naltrexone in treating opioid dependence is limited due to low patient compliance (6–8). Thus, developing long-term, sustained-release naltrexone formulations and addressing medication adherence is critical for effective relapse prevention.

In 2010, FDA approved a once-monthly injection regimen of sustained-release naltrexone (XR-NTX) for preventing drug relapse in opioid-dependent patients after detoxification (9, 10). However, the once-monthly injection did not completely resolve the issue of poor patient compliance, resulting in a relatively high dropout rate (11–13). Besides the injectable naltrexone depots, naltrexone implants with 3–6 months duration have also been investigated as a treatment for opioid dependence. An Australian-produced implant (O’Neil Implant, Go Medical Industries, Perth, Australia) consists of 10 pellets containing a poly-lactic-based polymer and naltrexone in dosages ranging from 1.1 to 1.8 g. The use of a single implant with 1.8 g naltrexone is found to release naltrexone above 1–2 ng/mL one month after surgery for about three months. Another two implant formulations containing 1 g of naltrexone (Wedgewood Implant; Wedgewood Pharmacy, Sewell, NJ, USA, and Prodetoxon, Fidelity Capital, Moscow, Russia) were found to release naltrexone with significant interindividual variation. To effectively address the poor compliance issue, it is essential to develop a longer-acting naltrexone formulation that maintains a stable plasma concentration (14, 15).

The long-acting naltrexone implant (NTX-IMP) used in the study contains a triple controlled-release system called “microparticle-matrix-coating” to achieve long-term and stable release. Drug molecules are embedded in a polylactic acid polymer matrix using patented sustained-release technology. The matrix has a membrane-controlled microparticle skeleton which forms an embedded support system to prevent sudden release. The coating film plays a crucial role in controlling the drug extravasation rate and exerts a lasting effect through various mechanisms such as membrane control and erosion. In the NTX-IMP, polylactic acid (PLA) serves as the main ingredient that acts as a scaffold and facilitates sustained release, thereby effectively regulating drug release. Due to its biodegradability, the final degradation products are H2O and CO2, which are highly safe. Therefore, it is widely used for medical purposes, including sutures, orthopedic surgery, and bone plates (16, 17).

In the Phase I study of NTX-IMP, a total of 36 participants completed the trial. Based on the pharmacokinetic data from our phase I study, the plasma concentration of naltrexone at 0.75 g dose was below 1 ng/mL between 4 to 15 days after implantation, and exceeded 1 ng/mL between 22 to 121 days after implantation. Both the 1.5g and 2.25g groups exhibited stable plasma naltrexone levels, where effective levels of 1ng/ml were achieved within 4 hours post-implantation and maintained for approximately 150 days thereafter. Moreover, the maximum concentration (Cmax) achieved was approximately 10ng/ml. Oral NTX tablets displayed significant variability in plasma levels, reaching a Cmax of 20ng/ml but rapidly declining to near-zero levels after only 16 hours following administration. Given that the time duration during which the plasma concentration of naltrexone was greater than 1 ng/ml was similar between the 1.5g and 2.25g groups, we chose 1.5g as a high dose in our phase II study. The *in vivo* metabolism of naltrexone implants aligns with linear pharmacokinetics within the dose range of 0.75 g to 2.25 g. Considering that the plasma concentration of 0.75 g dose did not reach the minimum effective dose during the first 4 to 15 days after implantation, two dose groups of 0.9g and 1.5g were designed for our phase IIstudy.

The current optimal approach for evaluating the antagonist effect of long-term naltrexone is through a challenge test, which involves administering opioid agonists like hydromorphone in ascending dosages. During the challenge test, both objective and subjective changes in participants are measured, including alterations in respiratory rate, pupil diameter, heart rate, and other subjective sensations. These measurements serve as indicators to determine the effectiveness of opioid blockade by NTX-IMP (18, 19).

This study aimed to assess the efficacy, safety, and pharmacokinetic profile of NTX-IMP in blocking opioid agonist effects, utilizing an opioid challenge test with hydromorphone injections. Additionally, the study sought to identify the more effective dosages of NTX-IMP for further clinical trials.

## Method

### Study design

This study employed a phase-2, single-center, randomized, parallel-group, double-blind, controlled trial. Subjects were divided into high- and low-dose groups based on Phase I study results, receiving single implantation of either 0.9 g or 1.5 g of the NTX-IMP. Their response to opioid agonist challenge was evaluated during each challenge period over 24 weeks to assess the level of opioid blockage (10, 20). The flow of participants is shown in the CONSORT diagram below. The Ethics Committee of the Second Xiangya Hospital approved this study.

### Participants

Participants met the following criteria: (1) Aged 18-65 years; (2) Had a history of weekly opioid dependence for at least one year; (3) Were in an abstinent stage for minimum 30 days with negative urine drug and naloxone challenge tests for opioids; (4) Weighed at least 45 kg for males and 40 kg for females; (5) Passed a screening within 2 weeks before the study, showing no significant medical history of heart, liver, kidney, digestive tract, nervous system diseases, severe psychiatric disorders, or metabolic abnormalities. Must have normal or clinically insignificant results in physical examinations including ECG, blood pressure, heart rate, respiration, and lab tests such as chest X-ray, complete blood count, urinalysis, liver and renal function, blood glucose, blood lipids, coagulation function, and other tests deemed necessary by the clinician; (6) Had no eye disease or optic neuropathy; (7) Were capable of effective communication with the investigator and could adhere to study regulations.

The patients in this study voluntarily accepted compulsory isolation for drug rehabilitation with the consent of the public security organ of the people’s government at the county level or the city divided into districts. According to the legal policy of China, for vulnerable groups such as patients in compulsory isolated drug rehabilitation centers, we need to obtain consent forms from the legal guardians of these participants. Therefore, the study commenced after obtaining informed consent from both participants and their legal guardians, who signed a written consent form.

Exclusion criteria for the trial were: (1) individuals with significant medical conditions or recent major surgery (within 4 weeks); (2) those with a history of vital organ diseases, blood disorders, bleeding tendencies; (3) Patients with chronic pain, epilepsy, severe mental and neurological diseases, or suicidal tendencies; (4) Individuals allergic to hydromorphone or naltrexone; (5) pregnant or lactating women; (6) those with immunodeficiency including a positive HIV test; (7) Individuals participating in other drug clinical studies within 3 months or currently participating in such studies.

Eligible subjects were required to have a positive and typical opioid response to hydromorphone in the baseline challenge test.

### Randomization

This study adopted a randomized double-blind design to ensure that the relevant researchers, and participants were blinded to the study groups. Randomization was performed using block randomization method. Participants were randomized into either the 0.9g or 1.5g naltrexone group, with 1:1 allocation. Randomization was performed through the creation of a randomization table by PLAN process in SAS 9.4 or above version. The research team that performed the challenge test and follow-up assessments remained blind to the treatment assignment.

### Intervention

The naltrexone implants were administered subcutaneously in the lower abdomen through a small incision under local anesthesia. Both groups received an equal number of pellet implants, so participants in the low-dose group also received extra blank implants consisting of polylactic acid (PLA) with glucose. The surgical wound was carefully monitored and observed, and sutures were removed after 7 days based on the progress of wound healing.

### Hydromorphone challenge test

Hydromorphone challenge for all participants occurred in Compulsory Isolated Drug Rehabilitation Center, Jingzhou Public Security Bureau across all sessions. Hydromorphone challenge was conducted on the baseline day before treatment (Day -7) and on Day 7, 30, 60, 90, 120, and 150 following NTX-IMP implantation by intramuscular administration of hydromorphone at ascending doses of 0 mg (placebo, normal saline solution), 3 mg, 4.5 mg, and 6 mg at 1hour intervals. The agonistic effect was evaluated at time points of 15 min, 30 min, 45 min, and one hour after each intramuscular injection during the examination; an all-placebo challenge sequence was randomly substituted on one of the post-randomization days for each subject as a control for expectancy effects.

The challenge tests would be discontinued under circumstances that: (1) a subject exhibited significant agonist effects, such as nausea, vomiting, hypotension, or bradypnea; or (2) there was a reduction in average pupil diameter by one-third within a single session compared to the previous session or less than 2 mm.

During the challenge, subjects were isolated to avoid mutual influence.

### Outcome

#### Primary outcomes (VAS score value of question “do you feel any drug effect?” and pupil diameter)

Six visual analog scales (VAS) questions were used to measure the extent and duration of opioid blockage following hydromorphone challenge (“Do you feel any drug effect?”, “How high are you right now?”, “Do you like this drug?”, “Does this drug have any positive effect?”, “Does this drug have any negative effect?”, and “Does this drug make you feel uncomfortable?”). These VAS questions were chosen based on their previously demonstrated sensitivity to the effects of opioid agonists and antagonists (21). VAS used a 0-100 numerical rating of antagonistic effect, with “0” indicating no feeling at all and “100” indicating extremely strong. These VAS questions were asked every 15 min within 1 h after each challenger injection. The score value of the first question was used as primary evaluation measure.

Pupil diameter, blood pressure, respiratory rate, heart rate, and skin temperature were measured 15 min before the first injection and 15 min, 30 min, 45 min, and 60 min after each injection throughout the process. Pupil diameter was pre-specified as the primary efficacy indicator. The pupils of the subjects were photographed under consistent ambient illumination and subsequently measured from the photographs; the average diameter (in millimeters) was obtained by combining both horizontal and vertical diameters.

#### Secondary outcomes (VAS score value for the other 5 questions)

Except for the first question, the score values of the other five VAS questions were used as secondary measures.

### Safety endpoint

The safety and tolerability observation measures during the study period included adverse events (including implant site reactions), physical examination, vital signs, electrocardiogram, and laboratory analyses.

Study termination criteria: (1) If any serious adverse event related to study drug occurs. (2) If any subject’s Alanine Transaminase (ALT) in hepatic function indexes exceeds 3 × upper limit of normal (ULN) and total bilirubin (T-BIL) exceeds 2×ULN and ALT still exceeds 3×ULN and T-BIL still exceeds 2×ULN. (3) If a significant agonist response occurs after the hydromorphone challenge, the subject will not undergo the challenge test at the subsequent challenge points but will continue to receive safety observation and pharmacokinetic blood sample collection.

### Pharmacokinetic assessment

Blood samples (4 ml each time) were collected from subjects at about 08:00 am before naltrexone implantation (Day 1) and on Day 3, 7, 12 (± 1), 15 (± 1), 22 (± 1), 30 (± 1), 37 (± 1), 45 (± 1), 52 (± 1), 60 (± 1), 67 (± 1), 75 (± 1), 82 (± 1), 90 (± 1), 97 (± 1), 105 (± 1), 112 (± 1), 120 (± 1), 127 (± 1), 135 (± 1), 142 (± 1) and 150 (± 1) after implantation to determine the plasma concentrations of naltrexone and its active metabolite 6-β-naltrexol. Blood samples were drawn into heparin sodium anticoagulated blood collection tubes using standard venipuncture techniques and centrifuged at 2-8°C, 1500 x g for 10 min. The plasma was separated and frozen for storage at or below -30°C. The concentrations of naltrexone and 6-β-naltrexol were determined by a validated high-performance liquid chromatography and tandem mass spectrometry method.

### Sample size

Due to the exploratory feature of this phase-2 trial and limited efficacy information, there is no statistical hypothesis made. The original target sample size was set as 30, with an allocation ratio of 1:1. A total of 57 opioid-dependent subjects in the abstinent stage were screened, and 31 subjects were randomized.

### Statistical method

Both primary and secondary efficacy indicators were analyzed by calculating the slopes of the time-effect (i.e. dose-response) functions of treatment periods (22, 23).

Pharmacokinetic parameter calculations were performed using the non-compartmental model (NCA) of Phoenix WinNonlin version 7.0; statistical analysis was performed using SAS version 9.4. The main pharmacokinetic parameters of naltrexone and 6-β-naltrexol included: AUC0-150d, AUC0-inf, Cmax, Tmax, Cavg,0-90d, Cavg,0-120d, and Cavg,0-150d; the main pharmacokinetic parameters of hydromorphone included AUC0-t, AUC0-inf, Cmax, Tmax, MRT, AUC0-4h and Cavg, 0-4h.

Due to the outbreak of COVID-19 during the study, lockdowns were implemented in most urban and community settings, resulting in missing challenge visits for some participants. To address this issue, the following methods were employed: 1. Baseline Observation Carried Forward (BOCF): Efficacy data from the baseline challenge test were used to fill in missing information; 2. Last observation carried forward (LOCF): If follow-up visits and efficacy data were unavailable due to the early withdrawal of the participants from the trial, efficacy data from the previous completed visit (after naltrexone implantation) were used to fill in the missing data. If the missing visit occurred after the baseline and there were post-baseline visits both before and after the missing visit, the average of the efficacy data from the visits before and after the missing visit was used for imputation.

## Results

### Subject characteristics and completion

The study took place between 8 December 2018 and 13 May 2020. A total of 57 opioid-dependent subjects in the abstinent stage were screened, and 31 subjects (54.39% screening success rate) were enrolled, including 16 subjects (15 males and 1 female) in the 0.9 g naltrexone group, and 15 subjects (12 males and 3 females) in the 1.5 g naltrexone group (**Table 1**). In the 0.9 g naltrexone group, 13 out of 16 (81.25%) individuals completed the study. Three participants withdrew prematurely due to a local adverse event, a significant agonist response to hydromorphone, and voluntary withdrawal respectively. In the 1.5 g naltrexone group, all subjects completed the study. The 31 enrolled subjects ranged in age from 23 to 53 years old with BMI ranging from 17.2 to 27.8 kg/m2. All of them were Han. Demographic and baseline characteristics are summarized in **Table 2**. The age, height, weight, and body mass index did not show significant differences between the two groups. The study completion status are shown in detail in **Table 3**.

**Table 1:** Participants information.

Participant number	Age/Gender	Assigned dose group	Informed consent signing date	Height (cm)	Weight (kg)	Nationality
N001	42/male	1.5 g	2018-12-08	168	60	China
N002	40/male	0.9 g	2018-12-08	171	64	China
N003	38/male	0.9 g	2018-12-08	174	72	China
N004	34/male	1.5 g	2018-12-08	175	64	China
N005	34/male	0.9 g	2018-12-08	161	57	China
N006	44/male	1.5 g	2018-12-08	166	62	China
N007	34/male	1.5 g	2018-12-08	176	86	China
N008	47/male	0.9 g	2019-04-17	170	62	China
N009	31/male	1.5 g	2019-04-17	174	65	China
N010	39/male	1.5 g	2019-04-17	167	69	China
N011	43/male	0.9 g	2019-04-17	175	60	China
N012	53/male	0.9 g	2019-04-17	163	66	China
N013	39/male	1.5 g	2019-04-18	168	56	China
N014	52/male	0.9 g	2019-06-12	172	65	China
N015	35/male	1.5 g	2019-06-12	164	65	China
N016	40/male	1.5 g	2019-06-12	182	68	China
N017	33/male	0.9 g	2019-06-12	180	60	China
N018	49/male	0.9 g	2019-06-12	166	59	China
N019	31/female	1.5 g	2019-06-13	155	55	China
N020	46/male	0.9 g	2019-08-28	170	57	China
N021	29/female	0.9 g	2019-08-28	162	55	China
N022	30/male	0.9 g	2019-10-23	165	70	China
N023	35/male	1.5 g	2019-10-23	176	73	China
N024	42/female	1.5 g	2019-11-01	174	52	China
N025	35/female	1.5 g	2019-11-24	165	47	China
N026	48/male	0.9 g	2019-11-22	176	59	China
N027	42/male	0.9 g	2019-11-22	172	59	China
N028	48/male	0.9 g	2019-11-23	160	52	China
N029	24/male	1.5 g	2019-11-24	176	60	China
N030	28/male	1.5 g	2019-11-27	188	80	China
N120	23/male	0.9 g	2019-11-27	178	68	China

**Table 2:** Summary of demographic and baseline characteristics of participants.

	0.9 g naltrexone group (N=16)	1.5 g naltrexone group (N=15)	Total (N=31)
Age			
Mean	40.9	35.5	38.3
SD	8.96	5.58	7.89
Median	42.5	35.0	39.0
Min,Max	23, 53	24, 44	23,53
Gender-n (%)			
Male	15 (93.8)	12 (80.0)	27 (87.1)
Female	1 (6.3)	3 (20.0)	4 (12.9)
Nationality-n (%)			
Han nationality	16 (100)	15 (100)	31 (100)
Others	0	0	0
Height(cm)			
Mean	169.7	171.6	170.6
SD	6.24	8.08	7.13
Median	170.5	174.0	171.0
Min,Max	160, 180	155, 188	155,188
Weight(kg)			
Mean	61.6	64.1	62.8
SD	5.54	10.27	8.14
Median	60.0	64.0	62.0
Min,Max	52,72	47,86	47,86
Body mass index(kg/m2)			
Mean	21.41	21.75	21.57
SD	2.012	2.799	2.390
Median	21.45	21.50	21.50
Min,Max	18.5,25.7	17.2,27.8	17.2,27.8

**Table 3:** Study completion status.

Visit	0.9 g Naltrexone					1.5 g Naltrexone				
	Missed visit rate	3 mg Hydromor	7.5 mg Hydromor	13.5 mg Hydromor	Placebo	Missed visit rate	3mg Hydromor	7.5mg Hydromor	13.5 mg Hydromor	Placebo
	% (n1/N)	% (n/N1)	% (n/N1)	% (n/N1)	% (n2/N)	% (n1/N)	% (n/N1)	% (n/N1)	% (n/N1)	% (n2/N)
Day – 7	/	100 (16/16)	12.5 (2/16)	/	/	/	100 (15/15)	/	/	/
Day 7	/	100 (16/16)	100 (16/16)	75 (12/16)	/	/	100 (15/15)	100 (15/15)	86.7 (13/15)	/
Day 30	6.3 (1/16)	100 (10/10)	100 (10/10)	100 (10/10)	31.3 (5/16)	/	100 (11/11)	100 (11/11)	100 (11/11)	26.7 (4/15)
Day 60	37.5 (6/16)	100 (8/8)	75 (6/8)	75 (6/8)	12.5 (2/16)	20 (3/15)	100 (7/7)	100 (7/7)	100 (7/7)	33.3 (5/15)
Day 90	43.8 (7/16)	100 (4/4)	75 (3/4)	75 (3/4)	31.3 (5/16)	33.3 (5/15)	100 (7/7)	100 (7/7)	100 (7/7)	20 (3/15)
Day 120	18.8 (3/16)	100 (13/13)	100 (13/13)	46.2 (6/13)	/	13.3 (2/15)	100 (13/13)	100 (13/13)	84.6 (11/13)	/
Day 150	25 (4/16)	100 (12/12)	100 (12/12)	33.3 (4/12)	/	/	100 (15/15)	100 (15/15)	66.7 (10/15)	/

Hydromor: Hydromorphone.

n1 represented the number of subjects with missing visits;

n2 represented the number of subjects receiving placebo challenge;

N1 represented the number of subjects receiving Hydromorphone challenge, that is, N1 = N – n1 – n2;

N represented the number of subjects enrolled in the corresponding Naltrexone dose group, and n represented the number of subjects who could receive this dose of Hydromorphone.

### The antagonistic effect of NTX-IMP on ascending doses of hydromorphone

**Figure 1:**
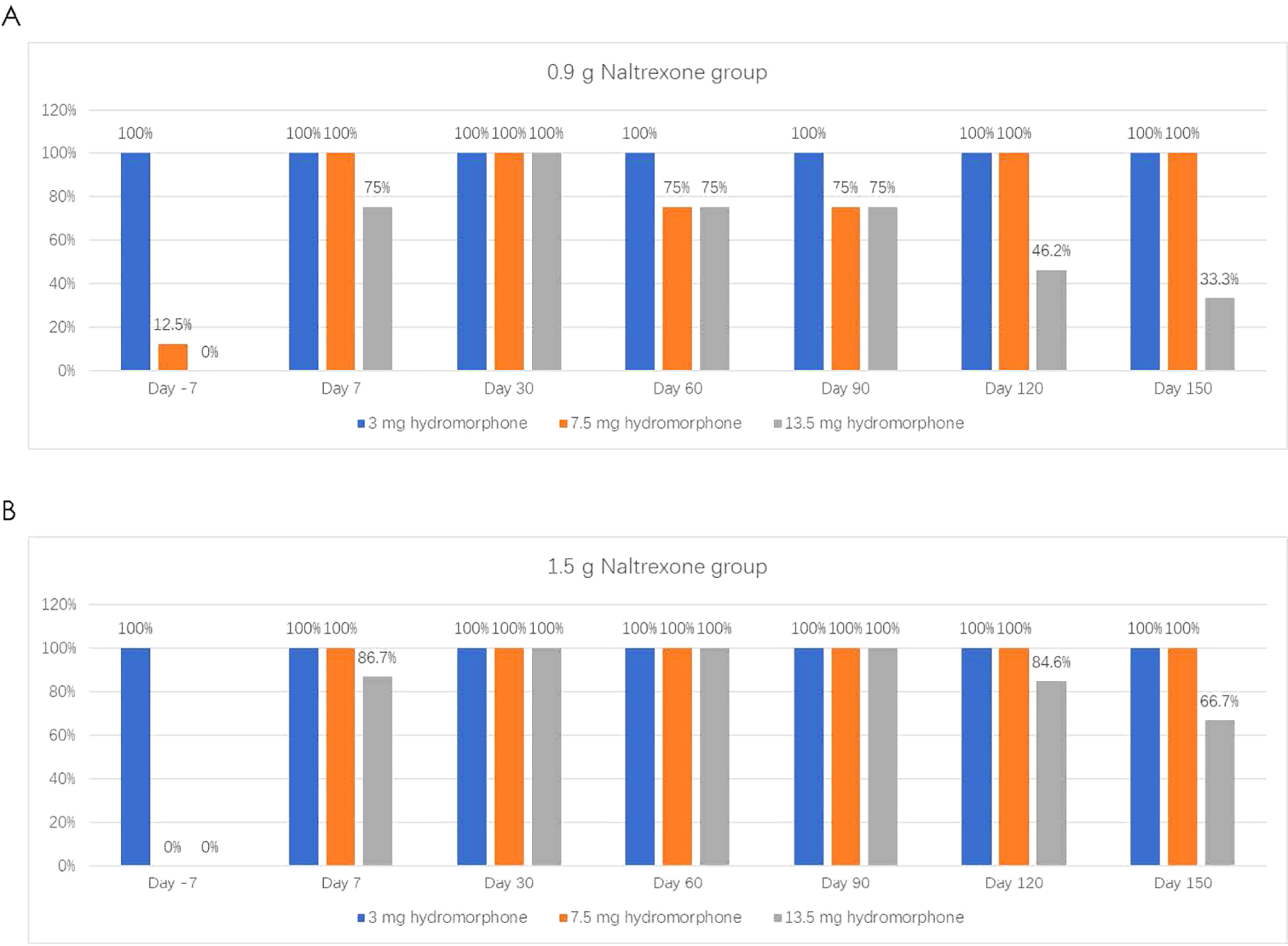
The proportion of subjects tolerating and receiving on different doses of Hydromorphone in the challenge tests in the 0.9g **(A)** and 1.5g **(B)** Naltrexone group. Hydromorphone challenge was conducted on the baseline day prior to treatment (Day -7) and on Day 7, 30, 60, 90, 120, and 150 following 0.9g **(A)** or **(B)** NTX-IMP implantation by intramuscular administration of hydromorphone at ascending doses of 0 mg (saline), 3 mg, 4.5 mg, and 6 mg sequentially at 1hour intervals. The subjects would not receive the next dose of hydromorphone when showing positive challenge results in the current challenge test.

**Figure 2:**
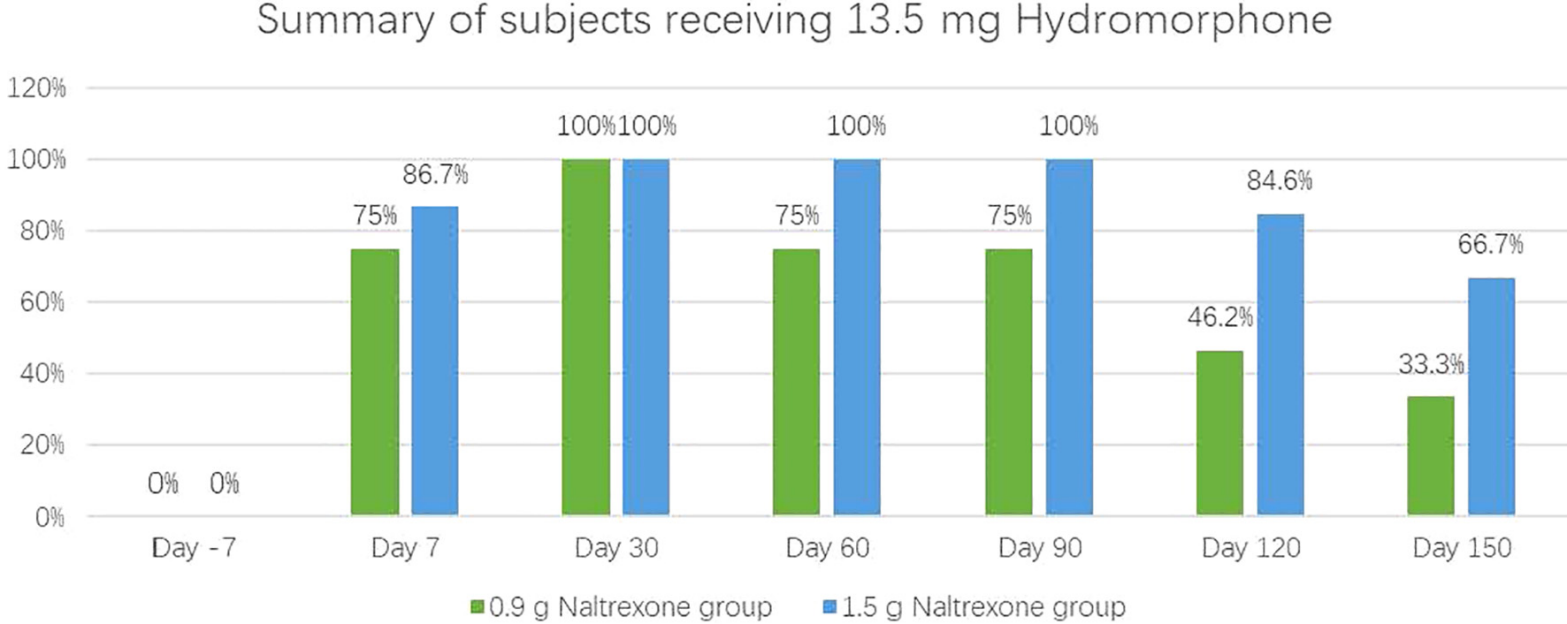
The proportion of subjects tolerating and receiving the maximum 13.5 mg cumulative hydromorphone in the 4-injection ascending dose sequence in 0.9 and 1.5 groups.

The intensity, onset, and duration of opioid blockage by NTX-IMP were assessed by whether the subject can tolerate hydromorphone challenge. **Figure 1** showed the proportions of subjects who exhibited tolerance to different doses of hydromorphone (i.e., challenge test negative) at various visit points in the 0.9 g and 1.5 g naltrexone groups respectively. After NTX-IMP implantation, the proportion of patients receiving different doses of hydromorphone in the 1.5g naltrexone group was generally higher than or equal to that in the 0.9g naltrexone group. The proportion of patients receiving 13.5mg hydromorphone was higher in the 1.5g naltrexone group (86.7% on day7, 100% on day60 and day 90, 84.6% on day120, 66.7% on day150) compared to the 0.9g naltrexone group (75% on day7, 75% on day60 and day 90, 46.2% on day120, 33.3% on day150) (**Figure 2**), showing that 1.5g naltrexone group had a stronger antagonistic effect.

Overall, these results indicated that NTX-IMP exhibited significant and sustained blockade of opioid agonist effects in a dose-dependent manner.

### Comparison of antagonistic effect by primary evaluation indicators

Two primary evaluation indicators (the score value of the VAS question(“Do you feel any drug effect?”) and pupil diameter) were included to compare the antagonistic effect between the two groups. We used linear regression to calculate the slope between the VAS score or pupil diameter and the dose of hydromorphone. The agonistic effect of hydromorphone was associated with a higher VAS score and smaller pupil diameter. If the slope difference was smaller than 0 for VAS scores or larger than 0 for pupil diameter, it indicated an antagonistic effect of naltrexone on hydromorphone.

**Table 4** showed the median of difference in slopes between each visit’s 3 mg hydromorphone challenge and baseline challenge. The median slope difference of the VAS score on day 7, 30, and 120 in the 0.9 g naltrexone group and day 7, 30, 60, 90, 120, and 150 in the 1.5 g naltrexone group, and of pupil diameter on day 7, 30, 60, 120, and 150 in the 0.9 g naltrexone group and day 7, 30, 60, 90, 120, and 150 in the 1.5 g naltrexone group, was statistically significant, suggesting a significant antagonistic effect of naltrexone on 3 mg hydromorphone.

**Table 4:** The median of difference in slopes between each visit’s 3 mg hydromorphone challenge and baseline challenge.

	0.9 g naltrexone group (N = 16)		1.5 g naltrexone group (N = 16)	
	Median slope difference (n)	P value	Median slope difference (n)	P value
“Do you feel any drug effect?” (score/min)				
Day 7	-0.059 (16)	<.001#	-0.135 (15)	<.001#
Day 30	-0.043 (10)	0.008#	-0.160 (11)	0.004#
Day 60	-0.022 (8)	0.688	-0.198 (7)	0.016#
Day 90	-0.048 (4)	0.375	-0.132 (7)	0.031#
Day 120	-0.037 (13)	0.005#	-0.114 (13)	<.001#
Day 150	-0.037 (12)	0.242	-0.102 (15)	<.001#
Placebo challenge visit	-0.051 (12)	0.004#	-0.134 (12)	<.001#
Pupil diameter (mm/min)				
Day 7	0.030 (16)	<.001#	0.031 (15)	<.001#
Day 30	0.032 (10)	0.002#	0.033 (11)	<.001#
Day 60	0.028 (8)	0.039#	0.028 (7)	0.016#
Day 90	0.026 (4)	0.25	0.033 (7)	0.016#
Day 120	0.016 (13)	0.003#	0.030 (13)	<.001#
Day 150	0.011 (12)	0.002#	0.029 (15)	<.001#
Placebo challenge visit	0.032 (12)	<.001#	0.033 (12)	<.001#

(1) The slope is determined using least squares linear regression. In this context, “x” represents the evaluation time points for assessing the antagonistic effect after intramuscular injection at each challenge time point, ranging from 15 to 120 minutes. “y” represents the VAS score and pupil diameter after intramuscular injection at each challenge time point.

(2) For non-placebo challenge visits, “n” represents the number of subjects challenged with 3 mg of hydromorphone, excluding those challenged with a placebo. For placebo challenge visits, “n” represents the total number of subjects participating in the placebo challenge.

(3) The p-value is obtained from the Wilcoxon signed-rank test, used to assess the difference in slopes between each visit’s 3 mg hydromorphone challenge and baseline challenge.

(4) “#” indicates a p-value less than 0.05, indicating a significant difference in slopes compared to the baseline challenge. If the slope difference for VAS scores is less than 0 and the slope difference for pupil diameter is greater than 0, it indicates an antagonistic effect of naltrexone on hydromorphone. If the difference is statistically significant, it suggests a statistically significant antagonistic effect of naltrexone on hydromorphone.

**Table 5** showed the median of difference in slopes between each visit’s full-sequence hydromorphone challenge and baseline challenge. The median slope difference of the VAS score on day 7, 30, and 120 in the 0.9 g naltrexone group and day 7, 30, 60, 90, 120, and 150 in the 1.5 g naltrexone group, and of pupil diameter on day 7, 30, 60, 120, and 150 in the 0.9 g naltrexone group and day 7, 30, 60, 90, 120, and 150 in the 1.5 g naltrexone group, was statistically significant, suggesting a significant antagonistic effect of naltrexone on full-sequence hydromorphone.

**Table 5:** The median of the difference in slopes between each visit’s full-sequence hydromorphone challenge and baseline challenge.

	0.9 g naltrexone group (N = 16)		1.5 g naltrexone group (N = 16)	
	Median slope difference (n)	P value	Median slope difference (n)	P value
Full-sequence - “Do you feel any drug effect?” (score/min)				
Day 7	-0.109 (16)	0.002#	-0.137 (15)	<.001#
Day 30	-0.074 (10)	0.004#	-0.146 (11)	0.002#
Day 60	-0.034 (8)	0.563	-0.198 (7)	0.016#
Day 90	-0.055 (4)	0.25	-0.133 (7)	0.031#
Day 120	-0.050 (13)	0.014#	-0.097 (13)	<.001#
Day 150	-0.048 (12)	0.193	-0.124 (15)	<.001#
Full-sequence placebo challenge visit	-0.091 (12)	0.004#	-0.145 (12)	<.001#
Full-sequence - Pupil diameter (mm/min)				
Day 7	0.026 (16)	<.001#	0.028 (15)	<.001#
Day 30	0.029 (10)	0.002#	0.029 (11)	<.001#
Day 60	0.027 (8)	0.039#	0.024 (7)	0.016#
Day 90	0.019 (4)	0.25	0.032 (7)	0.016#
Day 120	0.014 (13)	0.003#	0.029 (13)	<.001#
Day 150	0.009 (12)	0.005#	0.024 (15)	<.001#
Full-sequence placebo challenge visit	0.031 (12)	<.001#	0.033 (12)	<.001#

(1) The slope is determined using least squares linear regression. In this context, “x” represents the evaluation time points for assessing the antagonistic effect after intramuscular injection at each challenge time point, ranging from 15 to 240 minutes. “y” represents the VAS score and pupil diameter after intramuscular injection at each challenge time point.

(2) For non-”full-sequence placebo challenge visit”, “n” represents the number of subjects challenged with full-sequence hydromorphone, excluding those challenged with a placebo. For full-sequence placebo challenge visit, “n” represents the total number of subjects who received “full-sequence placebo challenge.”

(3) The p-value is obtained from the Wilcoxon signed-rank test, used to assess the difference in slopes between each visit’s full-sequence hydromorphone challenge and baseline challenge.

(4) “#” indicates a p-value less than 0.05, indicating a significant difference in slopes compared to the baseline challenge. If the slope difference for VAS scores is less than 0 and the slope difference for pupil diameter is greater than 0, it indicates an antagonistic effect of naltrexone on hydromorphone. If the difference is statistically significant, it suggests a statistically significant antagonistic effect of naltrexone on hydromorphone.

Secondary outcomes were evaluated by other five VAS questions. Similarly, linear regression was employed to calculate the slope between the VAS score and hydromorphone dose. The agonistic effect of hydromorphone was associated with a higher VAS score of questions “How high are you right now?”, “Do you like this drug?”, “Does this drug have any positive effect?”, and a lower VAS score of questions “Does this drug have any negative effect?”, and “Does this drug make you feel uncomfortable?”. The median slope of “How high are you right now?”, “Do you like this drug?”, “Does this drug have any positive effect?” during full-sequence hydromorphone challenge was higher in the 0.9g group than in the 1.5g group between 60 and 150 days after naltrexone implant treatment, indicating a larger antagonistic effect to hydromorphone challenge in the 1,5g group. The median slope of “Does this drug have any negative effect?”, and “Does this drug make you feel uncomfortable?” during 3mg hydromorphone challenge was lower in the 0.9g group than in the 1.5g group from 60 to 90 days and 7 to 90 days respectively after naltrexone implant treatment, indicating a larger antagonistic effect to 3mg hydromorphone challenge in the 1,5g group. However, during the full-sequence hydromorphone challenge, the median slope of these two questions was generally higher in the 0.9g group than in the 1.5g group, which may be due to the aversive response of participants and the small sample size (data not shown). A clinical trial among a larger population of patients will be conducted in the future.

### Pharmacokinetic parameters

**Figure 3:**
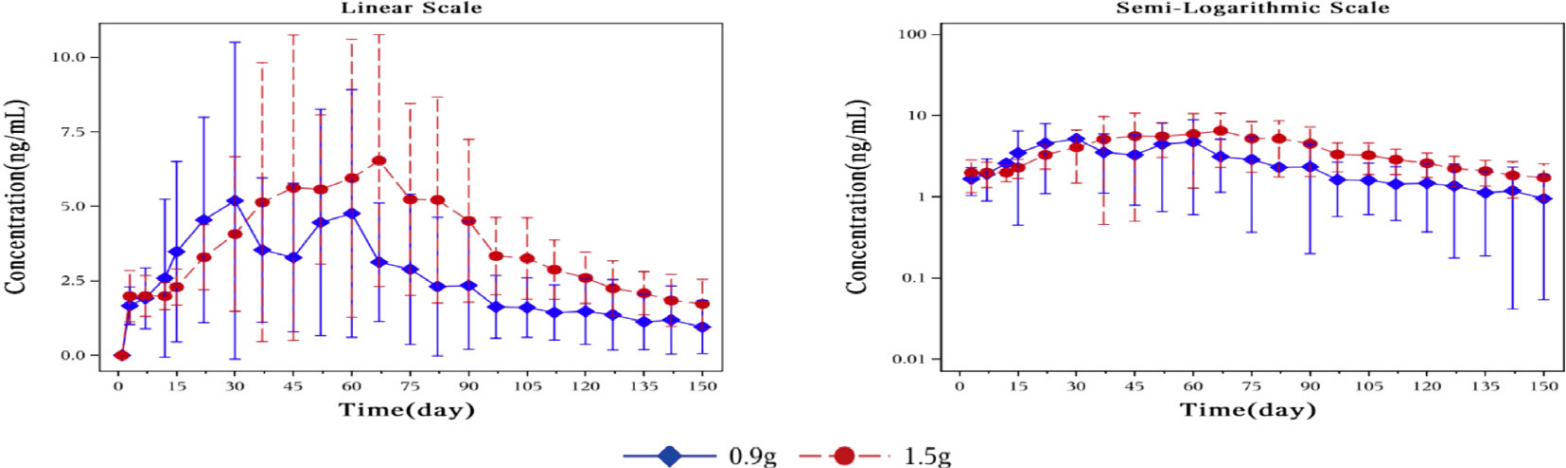
Mean plasma concentration of naltrexone over time for each dosage group on a linear scale (left) and semi-logarithmic scale (right). The blue rhombic points show the mean plasma concentration of naltrexone at different time points in the 0.9g naltrexone group. The red circular points show the mean plasma concentration of naltrexone at different time points in the 1.5g naltrexone group. Data are represented as mean ± SD.

**Figure 3** showed plasma concentrations of naltrexone at different time points. After implantation of 0.9 g and 1.5 g of naltrexone, the median Tmax was 50.45 days and 65.92 days, respectively. The Cmax was 9.0 ng/ml and 8.2 ng/ml, respectively, with no significant differences. **Table 6** showed that an increase in dose led to higher Cavg and AUC values.

**Table 6:** Summary of pharmacokinetic parameters of naltrexone.

Dose	Pharmacokinetics	n	Mean	SD	Median	Min	Max	CV%	GeoMean	CVb%
0.9 g Naltrexone (N = 16)	C_max_ (ng/mL)	14	8.9579	5.6716	7.3785	2.840	22.241	63.3	7.4312	71.7
	C_avg.0-90d_ (ng/mL)	11	3.3559	1.1290	3.3686	2.0382	5.7217	33.6	3.1937	33.7
	C_avg.0-120d_ (ng/mL)	11	3.0244	0.9018	3.0544	2.0233	4.8945	29.8	2.9102	29.4
	C_avg.0-150d_ (ng/mL)	6	2.5068	0.5158	2.5102	1.8152	3.0568	20.6	2.4610	21.5
	T_max_ (day)	14	/	/	50.45	10.94	72.89	/	/	/
	AUC_0-150d_ (day*ng/mL)	6	376.0167	77.3763	376.5293	272.2785	458.5251	20.6	369.1524	21.5
	AUC_0-inf_ (day*ng/mL)	6	454.2562	100.1439	458.0432	284.4588	599.0781	22.0	443.9893	24.6
0.9 g Naltrexone (N = 16) Sensitivity analysis	C_max_ (ng/mL)	11	7.4593	4.5491	5.5920	2.840	16.166	61.0	6.3230	66.1
	C_avg.0-90d_ (ng/mL)	11	3.3559	1.1290	3.3686	2.0382	5.7217	33.6	3.1937	33.7
	C_avg.0-120d_ (ng/mL)	11	3.0244	0.9018	3.0544	2.0233	4.8945	29.8	2.9102	29.4
	C_avg.0-150d_ (ng/mL)	6	2.5068	0.5158	2.5102	1.8152	3.0568	20.6	2.4610	21.5
	T_max_ (day)	11	/	/	50.96	12.96	72.89	/	/	/
	AUC_0-150d_ (day*ng/mL)	6	376.0167	77.3763	376.5293	272.2785	458.5251	20.6	369.1524	21.5
	AUC_0-inf_ (day*ng/mL)	6	454.2562	100.1439	458.0432	284.4588	599.0781	22.0	443.9893	24.6
1.5 g Naltrexone (N = 16)	C_max_ (ng/mL)	15	8.1868	6.1348	5.9480	2.882	23.444	74.9	6.6935	68.4
	C_avg.0-90d_ (ng/mL)	15	4.4924	1.8788	3.9721	2.4241	8.8960	41.8	4.1951	38.1
	C_avg.0-120d_ (ng/mL)	15	4.1533	1.3808	3.9341	2.4555	7.5941	33.2	3.9711	30.8
	C_avg.0-150d_ (ng/mL)	12	3.5573	1.0304	3.4448	2.3555	6.4142	29.0	3.4465	25.6
	T_max_ (day)	15	/	/	65.92	35.94	102.95	/	/	/
	AUC_0-150d_ (day*ng/mL)	12	533.5992	154.5646	516.7191	353.3176	962.1264	29.0	516.9700	25.6
	AUC_0-inf_ (day*ng/mL)	12	671.9300	191.2895	677.6370	428.5413	1000.8767	28.5	647.7634	28.8

Due to the early withdrawal of NO02 and NO20, incomplete collection of the absorption and elimination phase resulted in inaccurate calculation of PK parameters, therefore the descriptive statistics of PK parameters were not performed. The PK parameters from 3 subjects with abnormal plasma naltrexone concentration (test number: N003/N005/N011) were excluded for sensitivity analysis.

If the adjusted R2 is less than 0.8, the relevant parameters including AUC_0-inf_ are not accurate. If adjusted R2 is less than 0.8 and T_last_ is less than 150 days, AUC_0-150d_ and C_avg.0-150d_ are not accurate. If adjusted R2 is less than 0.8 and T_last_ is less than 120 days, C_avg.0-120d_ is also not accurate. If the adjusted R2 is less than 0.8 and the T_last_ is less than 90 days, C_avg.0-90d_ is also not accurate and treated as a missing value for analysis in the statistical summary.

**Figure 4:**
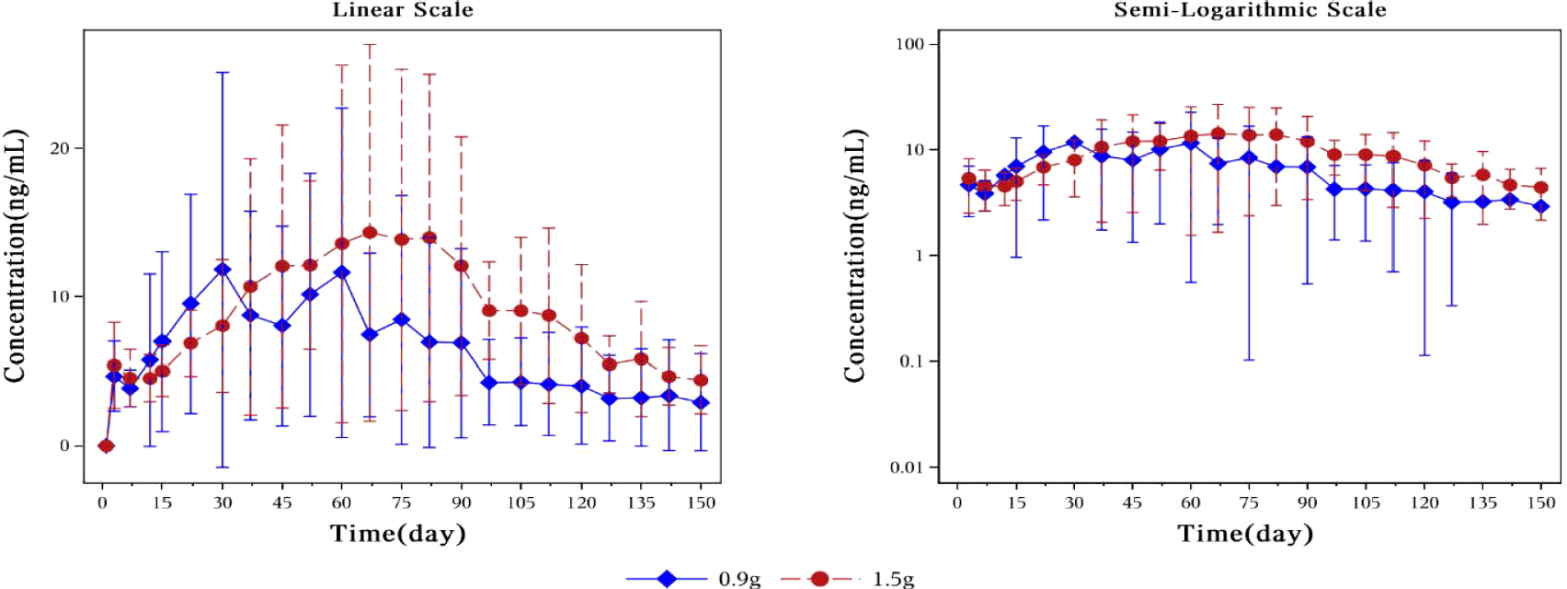
Plot of mean plasma concentration of 6-β-naltrexol at different time points from day 0 to day 150 on a linear scale (left) and semi-logarithmic scale (right). The blue rhombic points show the mean plasma concentration of 6-β-naltrexol at different time points in the 0.9g naltrexone group. The red circular points show the mean plasma concentration of 6-β-naltrexol at different time points in the 1.5g naltrexone group. Data are represented as mean ± SD.

The plasma concentration of naltrexone reached 1 ng/ml on the third day after implantation in all subjects except for one subject in the 0.9 g naltrexone group. The condition where plasma concentrations of naltrexone were ≥1 ng/ml lasted more than 148 days, with an average duration of 106.81 days in the 0.9 g group, lasted for more than 148 days, with an average duration of 138.80 days in the 1.5 g group (**Table 7**). **Figure 4** showed the median plasma concentrations of the major naltrexone metabolite, 6-β-naltrexol, at various time points. The results showed that the concentration of 6-β-naltrexol was initially twice that of naltrexone. Furthermore, the Cavg and AUC showed an increase with the dose, while the Cmax did not vary significantly. The concentrations were dose-dependent and gradually declined over time, reaching below the lower limit of quantitation after 150 days.

**Table 7:** Summary of statistical summary (%) of time to naltrexone plasma concentration ≥1 ng/ml.

Statistics	Results		
	0.9 g Naltrexone group	0.9 g Naltrexone group - sensitivity analysis	1.5 g Naltrexone group
N	16	11	15
Mean	106.81	137.27	138.80
CV%	49.66	16.33	21.32
SD	46.49	11.89	15.36
Min	20	110	68
Median	127	148	148
Max	148	148	148

### Adverse reactions

**Table 8** provided a summary of all adverse events that occurred during this study. Out of the 31 participants who received implants, 24 individuals experienced a total of 70 mild to moderate adverse events. Hepatitis C was an adverse event that resulted in remission, while other adverse events were resolved with time. Twelve adverse events that occurred before naltrexone implantation were assumed to be “definitely related” to hydromorphone injection. 58 adverse events occurred after naltrexone implantation, of which 24 (41.3%) were assumed to associate with naltrexone implants (possibly, probably, or definitely relevant), 15 (25.9%) were assumed to associate with hydromorphone (possibly, probably, or definitely relevant), and 19 (32.8%) were assumed to be “probably unrelated” to naltrexone implants. Adverse events associated with naltrexone implants occurred in 8 of 16 patients (50.0%) in the 0.9g naltrexone group and in 3 of 15 patients (20.0%) in the 1.5g naltrexone group.

**Table 8:** Summary of all adverse events.

	0.9 g naltrexone (N=16)		1.5 g naltrexone (N=15)		Total (N=31)	
	e	N (%)	e	N (%)	e	N (%)
Adverse events	41	13 (81.3)	29	11 (73.3)	70	24 (77.4)
Treatment-emergent adverse events (TEAEs) a	36	12 (75.0)	22	10 (66.7)	58	22 (71.0)
Adverse reactionsb	36	11 (68.8)	15	8 (53.3)	51	19 (61.3)
Related to hydromorphone Hydrochloride Injection	18	7 (43.8)	9	5 (33.3)	27	12 (38.7)
Related to naltrexone implant	18	8 (50.0)	6	3 (20.0)	24	11 (35.5)
Adverse reactions in TEAEs	31	10 (62.5)	8	5 (33.3)	39	15 (48.4)
Serious adverse event (SAE)	0	0	0	0	0	0
Serious adverse reactions	0	0	0	0	0	0
Adverse events leading to dropouts	1	1 (6.3)	0	0	1	1 (3-2)
Adverse reactions leading to dropouts	1	1 (6.3)	0	0	1	1 (3.2)

e=number of adverse events; n=number of subjects with adverse events; %=n/N*100. TEAE, treatment-emergent adverse event.

a) Treatment emergent adverse events (TEAEs) were defined as those arising after naltrexone implantation.

b) An adverse reaction refers to an adverse event judged to be definitely related, possibly related, and probably related to a drug.

For the calculation of the number and percentage of subjects with AEs, the same subject who experienced multiple occurrences of the same AE was counted only once. For AE cases calculated, the multiple times of same AE occurred more than once for the same subject was calculated.

Abnormalities in hepatic function were the most frequently observed adverse events, which was identified through the measurement of alanine aminotransferase, aspartate aminotransferase, and bilirubin. A total of 15 such events occurred in 10 subjects (32.3%) in the two groups, with all events being mild or moderate. Except for one case with hepatitis C infection, these events were classified as “recovered”, and the relationship with naltrexone implantation was deemed to be either “definitely related” or “possibly related”. In the 0.9 g naltrexone group, 7 subjects (43.8%) experienced 10 events in abnormal liver function, whereas in the 1.5 g naltrexone group, only 3 subjects (20.0%) experienced 5 such events.

Local reactions at the implantation site were observed in 2 subjects (12.5%) from the 0.9 g naltrexone group, and in 2 subjects (13.3%) from the 1.5 g naltrexone group, which were classified as grade 2 (significant) reactions. The remaining reactions were categorized as grade 1 (mild) or grade 0 (none), with no reports of grade 3 (severe) reactions. One subject (6.3%) from the 16 subjects in the 0.9 g naltrexone group experienced a local adverse event called “aseptic inflammation”, resulting in voluntary withdrawal. None of the 15 subjects in the 1.5 g naltrexone group discontinued participation due to any adverse events.

## Discussion

The main challenge in utilizing naltrexone for patients with opioid use disorder lies in ensuring compliance with oral preparation. Addressing the issue of poor medication adherence has become crucial for achieving effective relapse prevention using naltrexone. The study indicates that implantation of either 0.9 g or 1.5 g NTX-IMP exhibits significant and sustained opioid blockade for up to 150 days, with plasma concentrations exceeding the effective concentration threshold of naltrexone (1ng/ml) required for antagonizing opioid use (24). The enduring effect implies that long-acting naltrexone preparations exhibit significant efficacy in the treatment of opioid dependence.

The opioid agonist challenge doses used in this study have substantial clinical relevance. As a μ-opioid agonist, hydromorphone exhibits approximately 3-5 times greater potency than heroin (25). Wallenstein S.L. reported that intramuscular administration of hydromorphone was fivefold more effective than heroin at the same dosage in terms of analgesia, mood changes, and sleep. Additionally, its terminal elimination half-life after intravenous injection was estimated to be around 2.3 hours. In clinical pharmacology studies conducted in the United States on opioid addiction, hydromorphone has often been employed as a surrogate for heroin. An epidemiological survey by Tan et al. (26), which involved 2513 drug abusers, showed that the average daily dose of heroin abuse per individual in China was 0.68 g. However, due to various additives present in street heroin, the actual content of pure heroin generally fell below 10% (26). Assuming a content calculation of 10%, the average daily dose of pure heroin abused by drug users would equate to 68 mg or approximately 13.6 mg of hydromorphone. The total amount of hydromorphone administered within a three-hour period during this study design reached an equivalent level as the average daily dose abused by individuals with heroin dependence. Therefore, the challenge dose with hydromorphone designed for this study can effectively reflect the antagonistic effect exerted by naltrexone implants on patients with heroin dependence.

The US FDA’s drug review report on Vivitrol states that a minimum concentration of 1ng/ml naltrexone is needed to effectively block the opioid effects (27). Additionally, Hulse et al.’s study demonstrated that naltrexone implants were more effective than oral naltrexone in reducing psychological cravings and relapse rates among patients with heroin dependence. The study also found that plasma concentrations ranging from 1-3 ng/ml of naltrexone were associated with preventing psychological cravings and relapses, exhibiting a dose-dependent effect (28). Another study reported that naltrexone levels above 2 ng/mL blocked nearly all VAS ratings of drug liking after intravenous heroin administration (29).

In this study, following the implantation of 0.9 g and 1.5 g naltrexone in subjects, the average plasma concentration of naltrexone remained ≥1 ng/ml for over 148 days starting from approximately day 3. The population pharmacokinetic model showed that the 1.5g NTX group had significantly higher plasma concentration compared to the 0.9g group. In the 1.5g group, the duration of plasma concentration ≥2ng/mL lasted at least 130 days, while in the 0.9g group it lasted for 100 days. Similarly, a duration of ≥1ng/mL lasted at least150 days in the 1.5g group and 130 days in the 0.9g group. Additionally, there was a consistent correlation between NTX plasma concentration and its antagonistic effect on hydromorphone.

The study found that after implantation, four individuals in the 0.9 g naltrexone group experienced a rapid increase in plasma concentrations. The possible reason could be the addition of four blank implant pellets composed of polylactic acid (PLA) and glucose, resulting in an accelerated release of naltrexone. It is known that the glucose present in the blank implant pellets readily dissolves in water, facilitating rapid infiltration of tissue fluid into the scaffold. Additionally, PLA undergoes accelerated degradation due to main chain scission, leading to the formation of carboxyl termini and hydrolysis of ester groups within the long chains. This process generates oligomers and lactic acid monomer fragments, creating an acidic environment that further enhances PLA hydrolysis rate through autocatalysis. The autocatalytic effects can create a local acidic environment *in vivo* which may result in non-specific inflammation and accelerated drug release.

**Figure 5:**
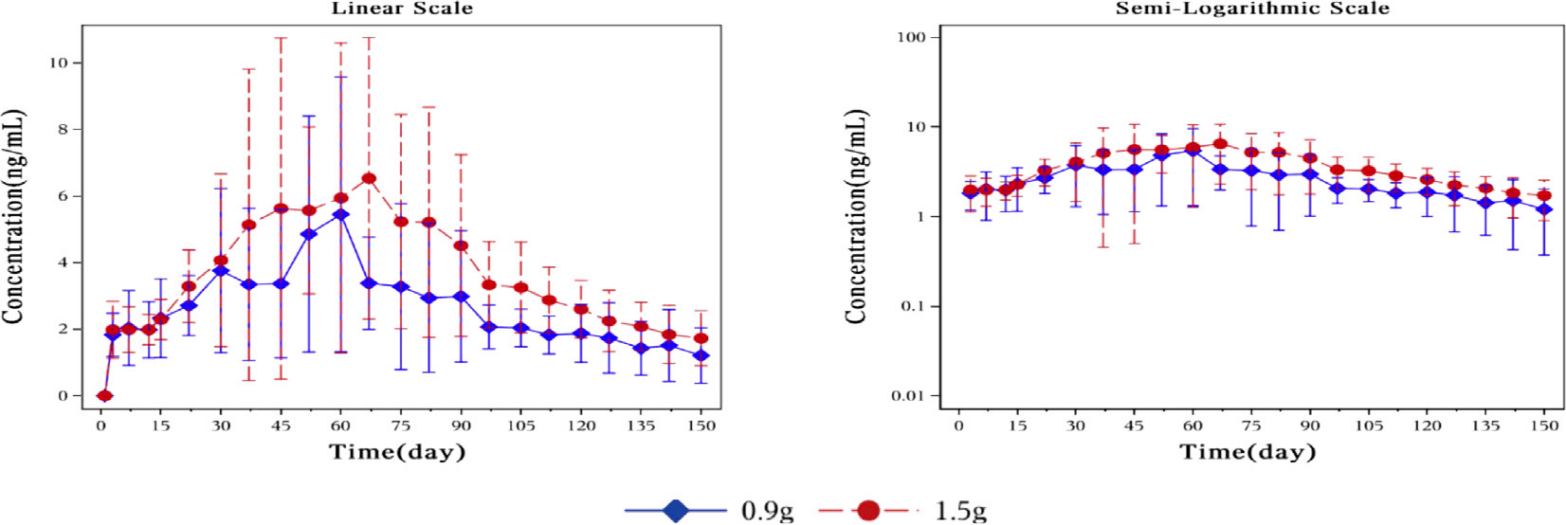
Mean plasma concentration of naltrexone over time for each dosage group in sensitivity analysis. Sensitivity analysis was conducted by excluding the blood drug concentration data of four subjects. The blue rhombic points show the mean plasma concentration of naltrexone after sensitivity analysis at different time points in the 0.9g naltrexone group. The red circular points show the mean plasma concentration of naltrexone after sensitivity analysis at different time points in the 1.5g naltrexone group. Data are represented as mean ± SD.

**Figure 6:**
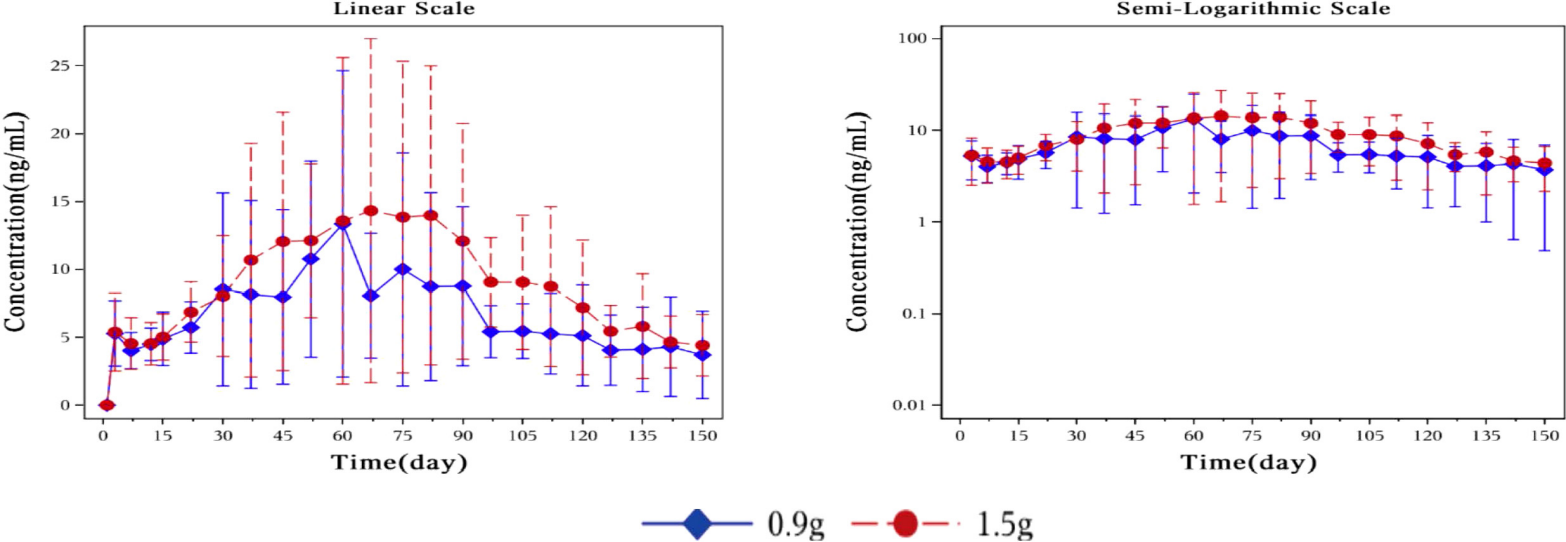
Plot of mean plasma concentration of 6-β-naltrexol after sensitivity analysis at different time points from day 0 to day 150 on a linear scale (left) and semi-logarithmic scale (right). Sensitivity analysis was conducted by excluding the blood drug concentration data of four subjects. The blue rhombic points show the mean plasma concentration of 6-β-naltrexol after sensitivity analysis at different time points in the 0.9g naltrexone group. The red circular points show the mean plasma concentration of 6-β-naltrexol after sensitivity analysis at different time points in the 1.5g naltrexone group. Data are represented as mean ± SD.(same above). The nonlinear mixed effect model was used to establish the population pharmacokinetic model of naltrexone implant. The final model was used to simulate the plasma concentration-time curve of two groups of doses of Naltrexone in 1000 subjects, as shown in the figure below: The nonlinear mixed-effects model was utilized to analyze the mean plasma concentration of naltrexone over time. The model was established by simulating the plasma concentration-time curve for two groups consisting of 1000 subjects.

Sensitivity analysis was conducted by excluding the plasma drug concentration data of four subjects (**Figures 5**, **6**). Compared to the drug concentration-time curve prior to exclusion, the average blood drug concentration in the 0.9 g naltrexone group during the first 30 days was significantly lower than that in the 1.5 g naltrexone group, with reduced intra-group differences.

The subjects reported good tolerance to the naltrexone implant, with most adverse events falling under the mild or moderate category and being deemed unrelated to the study drug. Furthermore, the outcomes were categorized as “remission” or “recovered”. Lastly, there were no serious (grade 3) implant site reactions observed during the study.

Many clinicians appear to express concerns regarding the potential hepatotoxicity of naltrexone, and some widely cited early studies have reported abnormalities in liver function tests among patients receiving high doses, particularly those with obesity. These concerns may be reinforced by recommendations in the product information sheet to conduct liver function tests before and during treatment, as well as the elevated rates of hepatitis C infection among patients with heroin dependence and the frequent occurrence of abnormal liver function test results in patients with alcohol use disorder. However, a retrospective study of 3285 patients with cirrhosis who were prescribed naltrexone suggested that naltrexone was not associated with the development of drug-induced liver injury and appeared to be safe in patients with compensated and decompensated cirrhosis (30). Another study, which performed a secondary analysis of data from a randomized clinical trial testing the efficacy of combined pharmacobehavioral harm-reduction treatment in improving alcohol and quality-of-life outcomes for adults experiencing homelessness and alcohol use disorder, suggested that receipt of extended-release naltrexone (XR-NTX) was not associated with hepatotoxicity (31). Our study findings demonstrate that all observed liver function abnormalities were mild to moderate in severity, with outcomes classified as “recovered”. Importantly, there was no proportional increase in adverse events associated with higher doses of naltrexone, indicating an absence of dose-related toxicity.

The limitations of this study should be acknowledged, including small sample size and potential inadequacy of hydromorphone doses for all patients with opioid use disorders. The results of challenge tests should be verified for their actual efficacy for relapse prevention of opioid dependence through clinical trials.

In summary, the results of this human study demonstrate the sustained pharmacological property of a long-acting naltrexone implant in effectively blocking or attenuating opioid agonist effects, thereby potentially preventing relapse in patients with opioid dependence in clinical practice. These findings provide further evidence and quantification of the safety profile associated with long-acting naltrexone implant.

## Conclusion

This study showed that naltrexone implant provides significant and sustained opioid blockade for up to five months, with plasma concentrations exceeding the effective concentration threshold of naltrexone required for antagonizing opioid use. The high-dose group exhibited superior antagonist efficacy compared to the low-dose group in hydromorphone challenge tests, indicating dose dependency. The NTX-IMP exhibits a safe profile with generally mild or moderate, and reversible adverse events. Our findings demonstrate that this new formulation of naltrexone has a promising efficacy and safety profile in clinical settings.

## Data Availability

The original contributions presented in the study are included in the article/supplementary material. Further inquiries can be directed to the corresponding author.
